# Professional, organizational and policy-level barriers and facilitators to perinatal mental health care in the United Arab Emirates: A qualitative study

**DOI:** 10.1371/journal.pone.0344312

**Published:** 2026-03-06

**Authors:** Rouwida ElKhalil, Preetha Menon, Hiba Adam, Rasha Bayoumi, Eny Qurniyawati, Emad Masuadi, Luai A. Ahmed, Rami H. Al-Rifai, Iffat Elbarazi

**Affiliations:** 1 Institute of Public Health, College of Medicine and Health Sciences, United Arab Emirates University, Al Ain, United Arab Emirates; 2 School of Psychology, Birmingham University, Dubai, United Arab Emirates; 3 Department of Epidemiology, Biostatistics, Population Studies, and Health Promotion, Faculty of Public Health, Universitas Airlangga, Surabaya, Indonesia; 4 Research group for health and wellbeing of women and children, Universitas Airlangga, Indonesia,; 5 Health Education Research Unit (HERU), Institute of Public Health, College of Medicine and Health Sciences, United Arab Emirates University, Al Ain, United Arab Emirates; University of Pretoria, SOUTH AFRICA

## Abstract

**Background:**

Perinatal mental health (PMH) is a critical component of maternal and child health, yet significant gaps persist in its integration into routine maternity care in the United Arab Emirates (UAE). Frontline healthcare professionals (HCPs) are central to identifying and addressing PMH concerns; however, their effectiveness is often hampered by multi-level barriers. This qualitative study aimed to identify the professional, organizational, and political-level barriers and facilitators influencing the implementation of PMH care from the perspective of HCPs in the UAE.

**Methods and findings:**

A descriptive qualitative design was employed. 43 HCPs, including lactation consultants, midwives, maternity nurses, obstetricians, family physicians, paediatricians, and psychiatrists/psychologists, were recruited using purposeful sampling. Data were collected through semi-structured interviews and focus group discussions. Thematic analysis was employed, and barriers and facilitators were categorized to identify key themes. The study identified 31 barriers and 33 facilitators across three ecological levels. Major barriers included PMH awareness and training gaps, low self-efficacy, role-based avoidance, fragmented services, staffing shortages, unclear protocols, and limited insurance coverage. Key facilitators encompassed professional development initiatives, core provider qualities like empathy and advocacy, interprofessional collaboration, integrated care models, supportive organizational policies, and government-led training programs. A critical finding was the role of the multicultural healthcare workforce as a significant facilitator for providing culturally competent care.

**Conclusion:**

The study identified multi-level barriers and facilitators that shape PMH care delivery in the UAE. Addressing these factors requires a systemic approach, including standardized PMH training, integrated care pathways, clear protocols, and policy reforms for insurance coverage. Leveraging the strengths of the multicultural workforce is essential for developing effective, culturally sensitive PMH services. These findings can guide the development of strategies to support HCPs and improve mental health outcomes for mothers and families.

## Introduction

Perinatal mental health (PMH), covering mental health issues during pregnancy and the first year postpartum, is a critical component of maternal and child health [1]. Left untreated, conditions like perinatal depression and anxiety can have serious consequences, including higher rates of preterm birth, low birth weight, impaired mother-infant bonding, and long-term developmental challenges for children [2,3]. Furthermore, PMH disorders are a significant contributor to maternal morbidity and mortality, with suicide being a leading cause of maternal death in high-income countries [4]. The impact extends beyond individual health, creating a substantial burden on families and society; economic evaluations reveal significant costs associated with increased healthcare utilization and reduced productivity [5]. Despite this prevalence and impact, PMH conditions frequently go unrecognized and untreated, underscoring an urgent need for improved screening and intervention strategies [6]

Frontline perinatal healthcare professionals, such as maternity nurses, midwives, lactation consultants, and obstetricians, are central to identifying and addressing PMH concerns As the primary point of contact for women during pregnancy and postpartum, they are uniquely positioned to detect early signs of mental distress and provide initial support or referrals [7]. However, their effectiveness is often hampered by multiple barriers, including insufficient mental health training, high workloads, time constraints, stigma, and unclear referral pathways [8,9]. Systemic challenges, such as poor integration between mental health and maternity services, a lack of standardized training, and cultural factors, further impede access to adequate care [10–12].

In the United Arab Emirates (UAE), these challenges are compounded by unique cultural and systemic factors. The healthcare workforce is overwhelmingly multinational, with approximately 97% of staff expatriates, introducing considerable linguistic and cultural diversity [13]. Although many providers possess international experience, formal cultural competence training is scarce, and self-perceived competence remains low [14]. Additionally, despite substantial national investment in maternal and child health, mental health services, particularly perinatal care, are often inadequately covered by insurance, especially under basic plans [15–17]. Referral pathways are also inconsistent and poorly coordinated, further limiting access to specialized PMH care [18]. Critically, there is a scarcity of qualitative evidence from the UAE exploring how frontline healthcare professionals perceive and navigate these barriers in their daily practice.

Given these complexities, a qualitative research approach is essential for developing a comprehensive understanding of the barriers and facilitators shaping PMH care delivery. Such approaches offer opportunities to explore healthcare professionals’ personal experiences and identify contextual and interpersonal factors that influence their practices. This method also highlights practical recommendations for education, training, and service delivery [19,20].

This qualitative study aimed to explore the multi-level factors influencing the implementation of PMH care in the UAE. Using the lived experiences and viewpoints of frontline healthcare professionals, it examined the barriers and facilitators at the professional, organizational, and political levels. The specific objectives were to:

1. Identify professional-level barriers and facilitators affecting healthcare professionals’ ability to provide PMH care
2. Explore organizational-level barriers and facilitators that shape PMH service delivery
3. Examine political- and policy-level factors influencing PMH care.

## Methods

### Design

This research utilized a descriptive qualitative methodology with interpretive thematic analysis to examine the perspectives of HCPs regarding the barriers and facilitators in perinatal mental health care. This methodology was selected due to its ability to support an abductive approach and foster interpretive interaction with established theoretical frameworks. [19,21]. To capture a comprehensive range of perspectives, data were collected through a combination of focus group discussions (FGDs) and semi-structured interviews. This methodological triangulation allowed for the exploration of both individual experiences and collective, interactive viewpoints within the perinatal healthcare context [22].

### Setting and sample

We conducted the study across three major UAE cities, Abu Dhabi, Dubai, and Al Ain, to ensure a diverse representation of healthcare settings and professional experiences. We recruited participants from May 1 to November 30, 2023, using purposive and snowball sampling. Recruitment occurred in various healthcare facilities (both outpatient and inpatient) and at educational events, such as Continuing Medical Education (CME) conferences.

Inclusion criteria mandated that participants: (1) be licensed HCPs (Registered nurses, midwives, lactation consultants, obstetricians, pediatricians, family medicine physicians, psychiatrists/ psychologists) holding at least a diploma; and (2) have a minimum of one year of active clinical experience. Recent graduates and HCPs with inactive professional status were excluded. Of the eligible HCPs approached, 43 agreed to participate, for a 73% participation rate; the primary reasons for declining were lack of interest in research and scheduling conflicts. Sampling continued until meaning saturation was achieved [23], with purposive efforts to ensure relatively equal representation across professional groups and inclusion of both public and private sector perspectives.

### Data collection

We collected data through two complementary methods: three virtual FGDs conducted via Microsoft Teams involving 15 healthcare professionals, and 28 semi-structured individual interviews, of which 9 were conducted face-to-face and 19 were conducted virtually via Microsoft Teams. The same semi-structured interview guide, piloted with two initial interviews and slightly refined, was used for both FGDs and interviews. The guide focused on participants’ perceptions, experiences, and the barriers and facilitators they encountered in providing PMH care (see Table 1). The interview guide, developed by the research team, focused on identifying barriers and facilitators to PMH practice as perceived and experienced by participants. Probing questions were used during interviews to clarify responses or to elicit more detailed information [24]. Minor modifications were made following two pilot interviews before the guide was applied to the remaining interviews. Each participant was interviewed only once. No prior relationships existed between the participants and the researchers. They were informed about the researchers’ background and motivations for the study. To ensure methodological consistency, two experienced qualitative researchers conducted all interviews. A reflexivity log was maintained to document how the team’s diverse professional backgrounds, including public health, nursing, and psychology, could influence data interpretation. Potential biases were actively managed through regular team discussions, which served to cross-check interpretations and ensure a balanced analytical process.

**Table 1:** Interview Guide.

Areas of Inquiry	Description	Semi-structure questions
Facilitators and Barriers in Perinatal Mental Health Practice	Exploring personal and organizational factors that enable or hinder perinatal mental healthcare professionals in implementing perinatal mental healthcare.	What are some of the personal challenges or barriers that maternal healthcare professionals face in providing perinatal mental healthcare? What personal strategies or practices have you found helpful in providing perinatal mental healthcare? Can you describe any specific organizational policies or practices that you feel are hindering the delivery of high-quality perinatal mental healthcare? Can you describe any policies or procedures in your workplace that support perinatal mental health practices?

Before the interviews, participants completed a brief demographic survey. The FGDs lasted 45–60 minutes, and individual interviews lasted 30–45 minutes. All sessions were audio-recorded and professionally transcribed; transcripts were then returned to participants for member checking to enhance accuracy. No substantial corrections were suggested, but participants confirmed the accuracy of the transcripts.

### Data analysis

We employed an inductive thematic analysis by Braun & Clarke [19] six-phase framework, facilitated by NVivo 14 software (QSR International™). The MATRIx framework is a multi-level conceptual model that synthesizes evidence on barriers and facilitators to implementing perinatal mental health care, categorizing influences at the professional, organizational, and policy levels [12]. The MATRIx framework did not inform data collection or act as a set of sensitizing concepts; rather, it was applied post hoc during data analysis to support the organization and interpretation of inductively derived themes.

The analytic process began with repeated reading and familiarization with the transcripts. Initial codes were generated inductively from the data and then systematically compared and mapped against the multi-level MATRix framework to situate findings across professional, organizational, and political levels of influence. This approach ensured that themes remained grounded in participants’ accounts while being contextualized within existing theoretical and empirical literature.

Themes from FGDs and interviews were initially analysed separately, then compared to identify convergences and divergences, enhancing analytic rigor. Codes were iteratively grouped into categories and refined into final themes. Coding was conducted independently by RE and PK. Any discrepancies were resolved through discussion, with a third researcher (IE) acting as an arbiter when necessary. The final phase involved defining themes and producing a detailed analytical report supported by participant quotes.

### Ethical approval

The Ethics Committee approved the study for Research in Social Sciences at the United Arab Emirates University (ERSC_2023_2749). Data collection commenced only after receiving ethical clearance. All participants were informed about the study aims, procedures, and their rights, including the option to participate voluntarily and to withdraw at any time without penalty. Written informed consent was obtained before each interview or focus group discussion.

To protect confidentiality, participants were asked not to disclose identifying information during focus groups, and pseudonyms were assigned during transcription. No personal identifiers appear in transcripts or reports. All data were securely stored and accessible only to the research team. The study adhered to the ethical principles of respect for persons, beneficence, justice, and compliance with applicable legal requirements. Participants who expressed interest were provided with information about freely accessible and paid resources to improve perinatal mental health literacy.

### Rigor

Several strategies were employed to ensure the study’s trustworthiness. The interview guide was reviewed and approved by two external experts. Techniques such as methodological triangulation (utilizing focus groups and interviews), analyst triangulation (involving multiple coders), and member checking (returning transcripts to participants) were employed. Furthermore, the research team, experienced in qualitative methods, engaged in regular discussions to cross-check interpretations and manage reflexivity. The study adhered to the COREQ checklist (COnsolidated criteria for REporting Qualitative research) to guide comprehensive reporting [25]. While frontline healthcare professionals were more frequently represented in relation to operational and clinical experiences, and mental health specialists contributed more system-level perspectives, no single professional group disproportionately shaped the themes, which reflected convergence across roles.

### Inclusivity in global research

Additional information regarding the ethical, cultural, and scientific considerations specific to inclusivity in global research is included in the Supporting Information (SX Checklist).

## Results

### Demographic data

The research involved 43 healthcare professionals (HCPs) from seven different specialties, with a predominance of females (90.7%) (Table 2). The participant pool was multinational, representing 14 different nationalities, including 14% UAE nationals, and 9.5% each from India, Pakistan, Jordan, and Palestine. The educational qualifications were notably high, with 86% of participants holding a bachelor’s degree or higher. The participants had significant experience, as 76.5% had 10 or more years in the healthcare field. Most HCPs (70%) were working within the public healthcare system. The average age of the participants was 41.70 years (SD = 10.05), and on average, they reported caring for an average of five maternal patients with mental health issues over the past year.

**Table 2:** Demographic Characteristics (*N = 43*).

Items	*n*	*%*
**Specialties**		
Family Medicine	6	14
Lactation Consultant	6	14
Registered Nurse	7	16
Obstetrician	6	14
Midwife	6	14
Psychiatric/Psychologist	6	14
Paediatrician	6	14
**Gender**		
Male	4	9
Female	39	91
**Nationality**		
UAE	6	14
Indian	4	9.5
Pakistan	4	9.5
Jordan	4	9.5
Sudan	3	7
Palestine	4	9.5
Egypt	3	6
Lebanon	2	5
South African	3	6
Syrian	3	6
Somalia	2	5
Poland	1	2.5
Filipins	2	5
France	1	2.5
Iraq	1	2.5
		
**Educational Level**		
Doctorate Degree	3	7
Master’s degree	16	37
Bachelor’s degree	21	49
Diploma Degree	3	7
**Working Experience**		
> 15 years	17	39.5
10 to 15 years	16	37
5 to 9 years	10	23.5
**Working Sector**		
Public	30	70
Private	13	30
	***Mean***	***SD***
**Age**	41.70	10.05
**Number of Maternal Patients with Mental Disorders Cared for in the Last Year**	5.51	0.68

### Thematic findings

The analysis of healthcare professionals’ viewpoints uncovered a complicated and diverse collection of factors that affect perinatal mental healthcare. The thematic results were categorized into three distinct yet interconnected ecological layers. A total of 31 barriers and 32 facilitators were recognized (Table 3, 4 and 5), offering a thorough insight into the obstacles that hinder and the supports that promote effective PMH practices. Importantly, a range of 10 dual factors, including foundational training, specialized PMH expertise, inter-professional collaboration, service design, integrated care models, screening systems, referral pathways, and sustainable financing, can act as either significant barriers or powerful enablers to the implementation of perinatal mental healthcare. The subsequent sections elaborate on these influential factors at the professional, organizational, and political tiers.

**Table 3:** Professional-level barriers and enablers to perinatal mental health care.

Barrier/ Facilitator	Themes	Subthemes	Illustrative Quotes
Barrier	**Theme 1:** PMH Awareness Gaps	**ST1:** Medication Safety Concerns	*− “There’s a general fear of medication related to pregnancy, not just among patients but also healthcare providers. This fear creates a stigma around prescribing medication.” (Psy3, II, Female)*
		**ST2:** Inability to Identify Red Flags	*− “You will not know if they have problems unless you will read [in the file] or they will announce.” (Obs2, II, Female)*
		**ST3:** Low Mental Health Literacy	*− “I don’t have that much deep knowledge about it [PMD]. It just self-learning whatever case I saw, I just go back and read.” (MW4, II, Female)*
		**ST4:** Undermining PMDs	*− “So sometimes the patient will not get even support from her own doctor, and these issues are undermined.” (LC6, FGD, Female)*
	**Theme 2:** Competency Gap Cycle	**ST1:** Limited Clinical Experience/Exposure	*− “Till now, I haven’t experienced such cases…” (Ped5, II, Male)* *− “I didn’t ever screen and refer. Never have I encountered this.” (MW1, II, Female)*
		**ST2:** Low Self-efficacy	*− “I wouldn’t be scared, but I would feel uncertain due to my lack of knowledge on how to manage their care.” (RN7, FGD, Female)* *− “I am embarrassed to say this, but I do not have much knowledge of this.” (Obs2, II, Female)*
		**ST3:** Role-based avoidance	*− “As a paediatrician or neonatologist, my focus is on the child. It’s challenging for me to address the mother’s well-being unless she is hospitalized and directly linked to obstetric care.”(Ped2, II, Female)* **−** *“We do not. But these tools are put into practice only by psychiatry, psychology...not by us.” (Obs2, II, Female)*
	**Theme 3:** PMH Training Gaps	**ST1:** Lack of Specialized Perinatal Mental Health HCPs	*− “We need professionals dedicated to this field [PMH] here in the UAE, and I believe this is still lacking.” (FM4, II, Female)*
		**ST2:** Educational Background of Healthcare Professionals	*− “Doctors who studied in the US or Canada tend to give more attention to psychology and psychiatry.” (Psy1, II, Female)*
		**ST3:** Limited Training in Perinatal Mental Health	*− “We need structured programs and training for primary healthcare doctors to effectively recognize and treat PMH conditions.” (FM3, II, Female)* *− “We don’t have training in this...As ob-gyn doctors and antenatal professionals, we should have some specific training related to mental health during the antenatal and post-natal.” (Obs6, II, Female)*
	**Theme 4:** Clinical Mental Health Engagement Barriers	**ST1:** Gender Dynamics in Care Provision	*− “As a male pediatrician, I find it challenging to shift focus from the infant to the mother, and patients’ acceptance of this can be an issue” (Ped3, II, Male)*
		**ST2:** Mental Health Communication Hesitation	*− “Staff are sometimes afraid to bring it up and say the word mental health to their patients.” (Psy4, II, Female)* *− “Even healthcare workers are hesitant to refer cases because they fear the patient and family will complain” (LC2, FGD, Female)*
		**ST3:** Language and Cultural Barrier	*− “This is a barrier, especially language barriers, such as when Arabic-speaking patients face difficulties communicating” (RN6, II, Female)* **−** *“We often face a language barrier...the questionnaire is unavailable in multiple languages” (MW6, FGD, Female)*
	**Theme 5:** Interdisciplinary Collaboration Issues	**–**	*− “Unfortunately, not all doctors cooperate. Only about two out of ten are willing to refer patients to me” (Psy1, II, Female)* *− “Only the staff who caring for that patient know that patient has that mental [health concern]...we don’t share the information” (MW4, II, Female)* **−** *“Not all psychiatrists want to take care of the pregnant patients” (Obs3, II, Female)*
Facilitator	**Theme 1:** Professional Development in PMH	**ST1:** Foundational Psychiatry Training	*− “As medical students, we have psychiatry rotations…it all comes with experience” (FM6, II, Female)* *− “During our residency, we received training on screening patients” (FM3, II, Female)*
		**ST2:** Specialization Opportunities	*− If we had subspecialties, I would prefer to focus on mental health” (FM2, II, Female)*
		**ST3:** Self-learning in Healthcare	*− “It’s self-learning…if you truly want to help women, you need to focus on improving yourself” (MW4, II, Female)* *− “I dedicate my time to studying mental health…With this expertise, I can detect, diagnose, and provide the best care” (FM2, II, Female)*
	**Theme 2:** Competency & Confidence	**ST1:** Communication Skills	*− “Be non-judgmental, listen actively, and maintain eye contact” (MW1, II, Female)*
		**ST2:** Cultural Competence	*− “Healthcare professionals should be culturally and linguistically skilled” (Psy4, II, Female)*
		**ST3**: Medication Competency	*− “We assess medication safety…and determine the best time for the mother to breastfeed” (LC6, FGD, Female)* *− “They come for prenatal care to adjust medications” (Obs1, II, Female)*
		**ST4:** Spotting Silent Struggles	*− At times, we observe infant neglect rather than obvious maternal mental health symptoms” (Ped3, II, Male)* *− “Lack of emotional support can be a red flag” (Ped1, II, Female)*
		**ST5:** Self-efficacy	*− “I’m not intimidated by dealing with these cases...they need the care” (MW1, II, Female)* *− “I’m confident because I have experience dealing with different families and cultures” (Ped4, II, Female)*
	**Theme 3:** Core Provider Qualities in PMH Care	**ST1:** Key Professional Attributes	*− “Empathy, active listening, and not being judgmental are very important” (FM4, II, Female)*
		**ST2:** Proactive Advocacy	*− “I took her to a family physician because the doctor refused to refer her” (LC6, FGD, Female)*
	**Theme 4:** Therapeutic Alliance and Trust-Building	**ST1:** Creating Confidential Spaces for Disclosure	*− “They hide that they are psychiatric patients, and you have to be patient with them” (FM6, II, Female)*
		**ST2:** Establishing Strong Rapport	*− “Making a good rapport with the patient makes a difference” (MW5, FGD, Female)* *− “She will open up if she trusts you” (Ped2, II, Female)*
	**Theme 5:** Interprofessional Synergy in a Multicultural Context	**ST1:** Diverse Professional Backgrounds	*− “Having coworkers from different backgrounds provides a broader perspective” (MW4, II, Female)* *− “A multicultural team helps provide perspective into the woman’s culture” (MW4, II, Female)*
		**ST2:** Collaboration Between HCPs	*− It was a multidisciplinary team effort” (FM2, II, Female)* *− “There is strong cooperation between obstetric and psychiatric departments” (Obs1, II, Female)* *− “We communicate with a psychiatrist; it’s a combined effort” (Obs6, II, Female)*

*ST = Subtheme; PMH = Perinatal Mental Health; II = Individual Interview; FGD = Focus Group discussion; Obs = Obstetrician; FM = Family medicine physician; RN = Registered Nurse; MW = Midwife; LC = Lactation consultant; Ped = Pediatrician; Psy = Psychiatrist/Psychologist*

**Table 4:** Organizational-level barriers and enablers to perinatal mental health care.

Barrier/ Facilitator	Themes	Subthemes	Illustrative Quotes
Barrier	**Theme 1:** Service Access Barriers	**ST1:** Service Location	*− “So, having clinics outside the hospital can help some categories of patients, but at the same time, being part of a team working inside a hospital will break the stigma a little bit” (Psy6, II, Female)*
		**ST2:** Service Availability	*− “The problem is the long waiting times. Sometimes, it takes up to two months for them to be seen, which is too long for patients. And I think it’s because there are a lot of patients at these two hospitals” (MW2, FGD, Female)*
		**ST3:** Logistical Challenges	*− “We need to arrange transportation or a consultation and have someone come to be with her” (MW4, II, Female)*
	**Theme 2:** Policy & Protocols barriers	**ST1:** Lack of Standardized Screening	*− “Screening is not a common practice here because most patients will be fine... However, screening becomes necessary when there is suspicion” (Obs5, II, Male)* *− “I didn’t ever screen and refer” (MW1, II, Female)*
		**ST2:** Lack of Case Management Guidelines	*− “We don’t have a clear protocol for case management as we do for other conditions” (Obs2, II, Female)*
		**ST3:** Unclear Referral Protocols	*− “I was asking myself: where should I refer?...I asked around” (FM5, II, Female)*
		**ST4:** Lack of Prioritization for Perinatal Mental Health	*− “So far I didn’t see an organization making a move regarding mental health for the perinatal” (MW4, II, Female)* *− “This is not really our priority” (RN7, II, Female)*
	**Theme 3:** Staffing and Workload Barriers	**ST1:** Staff Shortages	*− “Shortage of midwives in our facility makes it difficult to provide continuous care for patients, so we are only involved during labor” (MW5, FGD, Female)*
		**ST2:** High Patient Loads	*− “The doctor manages 15–17 patients, making the workload too high, which increases the risk of missing patients” (MW4, II, Female)*
		**ST3:** Time Constraints	*− “Time is a challenge, especially in busy healthcare centers today. Continuity of care becomes difficult when patients struggle to reach you. Appointment times are not protected, and frequent interruptions limit consultation time” (FM3, II, Female)*
	**Theme 4:** Fragmented and Under-Resourced PMH Services	**ST1:** Lack of Integrated Psychiatry	*− “There is no easy access for me as a healthcare provider to reach a psychiatrist and discuss the case with them” (FM3, II, Female)*
		**ST2:** No Dedicated Centers	*− “There is no specific center dedicated to addressing perinatal mental disorders with professionals who are specially trained” (FM4, II, Female)*
		**ST3:** Service Limitations	*− “The postnatal wards aren’t equipped to handle severe cases, like mothers attempting suicide” (Obs3, II, Female)*
	**Theme 5:** Uncoordinated Care Pathways	**–**	*− “The lack of regulation for life coaches can lead to wasted time and delays in accessing proper mental health care, as they lack the specialized training required for effective treatment” (Psy3, II, Female)*
Facilitator	**Theme 1:** Staffing Models	**ST1:** Support Staff Roles	*− “Social workers play a crucial role in domestic violence cases by assisting OB-GYN doctors and helping patients understand their rights” (Psy3, II, Female)* *− “We will try to contact the patient relation officer, social worker, and obstetric doctors to maybe put a referral for her for psychiatry” (Ped2, II, Female)*
		**ST2:** Dedicated Specialists	*− “Mental health support should include a dedicated team, such as a mental health nurse, social worker, or case manager… just as chronic disease clinics have specialized nurses” (FM2, II, Female)* *− “Having a counselor available daily would allow emotionally distressed patients to be referred for support” (Psy3, II, Female)*
	**Theme 2:** Hospital Services & Infrastructure	**ST1:** Hospital Services Design	*− “We are discussing a lot of possibilities about this idea of being creative in terms of service” (Psy4, II, Female)*
		**ST2:** Integrated Care Models	*− “We are definitely moving in the direction of the idea of an integrated care model” (FM3, II, Female)* *− “I believe it is crucial to have a psychologist and a social worker within the organization or primary healthcare center for patient referrals” (FM3, II, Female)*
		**ST3:** Specialized Mental Health Services	*− “We have psychiatrists and psychologists available in our clinics, though not daily…we can refer patients directly to the psychiatrist” (FM6, II, Female)* *− “If we had a direct psychiatry facility available in our hospital, maybe the process would have been quicker, easier, more convenient” (Obs2, II, Female)*
	**Theme 3:** Care Coordination & Continuity	**ST1:** Multidisciplinary Collaboration	*− “The clinic’s social worker at our Primary Healthcare stepped in, calling the patient and providing support” (FM2, II, Female)* *− “Therefore, we must work together in the maternity health facility. Not only do we need a psychiatrist, but also a psychologist and a social worker” (Psy3, II, Female)*
		**ST2:** Continuity of Care	*− “Providing continuous care, from antenatal to delivery, makes a significant difference” (MW5, FGD, Female)* *− “If there is an issue, then this issue would be more likely to be picked up on frequent visits” (Obs5, II, Male)*
		**ST3:** Extended Care Models (Perinatal Home Visit)	*− “Routine visits should include a specialist…who can assess the patient and, if needed, refer her to a higher level of care if at risk” (Ped4, II, Female)*
	**Theme 4:** Policy & Structural Support	**ST1:** Organizational Mental Health Framework	*− “The organization needs to implement a clear mental health policy” (MW4, II, Female)*
		**ST2:** Mental Health Awareness & Education	*− “Elevate mental healthcare to the same level of importance as other medical disciplines” (Obs2, II, Female)* *− “Education, education, and education...for the family and the patient” (Obs1, II, Female)*
		**ST3:** Service Accessibility	*− “If we do it routinely...we apply tools, and then we identify” (Obs2, II, Female)* *− “Screening tools should be key performance indicators” (FM3, II, Female)* *− psychiatric clinics in primary healthcare settings helps reduce stigma” (FM3, II, Female)*
		**ST4:** Standardized Screening Systems	*− “Only those with special access can read these [psychiatric] notes” (FM2, II, Female)*
		**ST5:** Electronic Health Records (EHRs)	*− “There should be a specific number and clear instructions on how to refer” (Obs5, II, Male)* *− “We need to track what happened to the patient after referral” (Obs2, II, Female)*
		**ST6:** Clear Referral Guidelines & Pathways	*− “There should be a specific number and clear instructions on how to refer” (Obs5, II, Male)* *− “We need to track what happened to the patient after referral” (Obs2, II, Female)*
	**Theme 5:** Workforce support	**ST1:** Mental Health Resources for HCPs	*− “We discussed setting up a hotline for OB-GYN doctors, along with an on-call arrangement and addressing various case management matters” (Psy3, II, Female)*
		**ST2:** Staff Wellbeing Programs	*− “The staff well-being program offers support therapy and relaxation sessions without documentation to encourage participation” (Psy2, II, Female)*

*ST = Subtheme; PMH = Perinatal Mental Health; II = Individual Interview; FGD = Focus Group discussion; Obs = Obstetrician; FM = Family medicine physician; RN = Registered Nurse; MW = Midwife; LC = Lactation consultant; Ped = Paediatrician; Psy = Psychiatrist/Psychologist.*

**Table 5:** Political-level barriers and enablers to perinatal mental health care.

Barrier/ Facilitator	Themes	Subthemes	Illustrative Quotes
Barrier	**Theme 1:** Financial and Insurance Constraints	**–**	*− “Patients will struggle, often being referred elsewhere with the burden of extra costs. Many services aren’t covered, so even those in need may not have insurance support to see a psychiatrist, psychologist, or other mental health professionals” (FM5, II, Female)* *− “Similarly, they might not be willing to pay for mental health care if it is not covered by insurance, especially when they have basic insurance plans” (Ped1, II, Female)* *− “Decreasing the fees is a step, but it’s not enough. There should also be exemptions available at hospitals. We are seeing more and more patients who don’t have insurance, and this issue needs more attention” (MW2, FGD, Female)*
Facilitator	**Theme 1:** Policy-Level Investment in Capacity and Access	**ST1:** Bridging Service Gaps through Policy-Driven Training	*− “This upskilling program is available in government and private hospitals through the Department of Health. It’s an ongoing initiative, currently in its fourth or fifth year, and continues to be improved and expanded annually” (Psy3, II, Female)*
		**ST2:** Access to Affordable Mental Health Services	*− “There needs to be a reduction in fees or an increase in insurance coverage to make services more accessible” (MW2, FGD, Female)*

*ST = Subtheme; PMH = Perinatal Mental Health; II = Individual Interview; FGD = Focus Group discussion; FM = Family medicine physician; MW = Midwife; Ped = Paediatrician; Psy = Psychiatrist/Psychologist.*

### HCP-level barriers

#### Theme 1: PMH Awareness Gaps.

The findings reveal gaps in HCPs’ PMH awareness, which critically affect how concerns are identified and managed. This theme is divided into four key subthemes. First, medication safety concerns reflect a general reluctance and fear of prescribing psychiatric medications during pregnancy, contributing to stigma around medication use. Second, the inability to identify red flags refers to symptoms that are often missed or misattributed due to uncertainty, particularly when patients do not explicitly disclose their concerns. Third, low mental health literacy highlights a lack of knowledge and familiarity with PMH concepts, with providers relying largely on self-directed learning when encountering PMH cases. Finally, undermining PMDs reflects a pattern of downplaying or minimizing the importance of mental health issues, sometimes resulting in limited support for affected women.

#### Theme 2: The Competency Gap Cycle.

This theme illustrates a self-reinforcing pattern of inaction among healthcare providers, which is closely tied to underlying awareness gaps. It begins with limited clinical experience and exposure, where infrequent encounters with PMH cases lead to uncertainty. This lack of exposure directly contributes to low self-efficacy, reflected in reduced confidence in addressing PMH concerns, even when some training is available. Ultimately, this manifests as role-based avoidance, in which providers distance themselves from PMH responsibilities and defer care to other disciplines, often perceiving PMH management as outside their professional scope.

#### Theme 3: PMH Training Gaps.

Further compounding these challenges are PMH training gaps, with healthcare providers widely acknowledging systemic and educational deficiencies in their preparation to manage PMH conditions. A critical issue is the shortage of specialized PMH professionals. Educational background was also reported to influence providers’ attention to mental health, with variability depending on training context. Across specialties, participants highlighted limited structured training in PMH, particularly related to initiating treatment and managing care during the antenatal and postnatal periods.

#### Theme 4: Clinical Mental Health Engagement Barriers.

Beyond knowledge gaps, providers faced significant clinical mental health engagement barriers that hindered their ability to initiate conversations about mental health. These challenges stemmed from complex social dynamics and personal discomfort, often preventing open dialogue. Gender-related expectations particularly affected male providers, influencing patient acceptance and provider confidence. Reluctance to initiate mental health discussions was common due to concerns about stigma, patient reactions, and potential complaints. Language and cultural barriers further complicated patient–provider interactions, limiting the effectiveness of screening tools and reducing opportunities for disclosure, even in settings where interpreter services were available.

#### Theme 5: Interdisciplinary Collaboration Issues.

Limited interdisciplinary collaboration emerged as a significant barrier to PMH care, reflecting fragmented communication and limited coordination in managing PMH cases. Providers described inconsistent cooperation between disciplines, reluctance to refer patients to mental health specialists, and breakdowns in information sharing during care transitions. These coordination gaps compromised continuity of care and contributed to fragmented management of PMH concerns.

### HCP-level facilitators

#### Theme 1: Professional Development in PMH.

Building on the identified training gaps, professional development in PMH emerged as a critical facilitator, highlighting how healthcare professionals actively strengthen their PMH expertise. This included foundational psychiatric training, residency exposure, continuous professional development activities, and self-directed learning. Participants emphasized the value of refresher courses, workshops, webinars, and opportunities for specialized training pathways to improve PMH care.

#### Theme 2: Competency and Confidence.

Healthcare professionals consistently emphasized that competency and confidence are equally vital for effective PMH care. Strong communication skills were viewed as essential for building therapeutic relationships. Cultural competence was particularly important within the UAE’s diverse healthcare context. Clinical competencies, including medication management during pregnancy and breastfeeding, were highlighted as key enablers. Providers also stressed the importance of identifying less obvious signs of mental distress and addressing psychosocial vulnerabilities. Increased self-efficacy enabled providers to engage more confidently in PMH care.

#### Theme 3: Core Provider Qualities in PMH Care.

Core provider qualities were identified as essential for effective PMH care, including empathy, active listening, and non-judgmental attitudes. Trust was described as developing through consistent and compassionate engagement over time. Providers also highlighted the importance of proactive advocacy and taking initiative to support patients when system-level barriers limited access to care.

#### Theme 4: Therapeutic Alliance and Trust-Building.

The foundational importance of a strong therapeutic alliance and trust-building emerged as a critical facilitator. Creating a safe, confidential, and supportive environment enabled patient disclosure and engagement. Establishing rapport was viewed as central to fostering trust, with repeated interactions strengthening relationships and increasing the likelihood of identifying mental health needs across the perinatal period.

#### Theme 5: Interprofessional Synergy in a Multicultural Context.

A key finding was the critical role of interprofessional synergy in a multicultural context, where the combined strengths of a diverse workforce and effective collaboration enhanced PMH care delivery. Multicultural healthcare teams supported culturally sensitive and contextually informed care. Meaningful collaboration between disciplines enabled shared expertise, coordinated treatment, and comprehensive support for women with complex PMH needs.

### Organization-level barriers

#### Theme 1: Service Access Barriers.

The study identified several critical service access barriers that create organizational challenges in delivering effective PMH care. These barriers primarily relate to service location, availability, and logistical constraints. Regarding service location, participants highlighted the complexity of clinic settings, noting that while services located outside hospitals may benefit some patients, hospital-based teams may help reduce stigma. Turning to service availability, long wait times and delays in receiving care emerged as a major concern, often attributed to high patient volumes within facilities. Additionally, logistical challenges further complicated access, including the need to arrange transportation, consultations, or patient accompaniment.

#### Theme 2: Policy and Protocol Barriers.

Beyond access issues, policy and protocol barriers represented another significant layer of organizational challenges. These systemic deficiencies affected screening, case management, and referral processes. Participants noted the lack of established screening procedures, with screening often occurring only when concerns were suspected rather than as routine practice. The absence of clear case management guidelines was equally problematic. Referral pathways were also described as unclear, with providers reporting uncertainty about where and how to refer patients. More broadly, participants highlighted institutional neglect of PMH, citing a lack of dedicated policies, resources, and prioritization within organizations.

#### Theme 3: Staffing and Workload Barriers.

Compounding these challenges were staffing and workload barriers. Shortages of trained professionals were identified as a key constraint affecting continuity and quality of care, particularly limiting involvement beyond labour and delivery. High patient volumes exacerbated these issues, leaving providers with limited time for individual cases and increasing the risk of missed concerns. Time constraints further hindered continuity of care, with busy clinical environments, unprotected appointment times, and frequent interruptions limiting meaningful engagement.

#### Theme 4: Fragmented and Under-Resourced PMH Services.

The fragmentation and under-resourcing of PMH services presented additional systemic obstacles. Limited access to integrated psychiatric support made it difficult for healthcare professionals to consult specialists and discuss complex cases. The absence of dedicated perinatal mental health centres further hindered care quality. Service limitations were particularly evident in severe cases, with postnatal wards described as insufficiently equipped to manage high-risk mental health presentations.

#### Theme 5: Uncoordinated Care Pathways.

Uncoordinated care pathways further exacerbated organizational challenges. The lack of structured collaboration between health and other service providers contributed to delays and gaps in recognition, referral, and management of mental disorders. The involvement of non-specialists, such as life coaches or religious healers, was reported to delay access to appropriate psychiatric care.

### Organization-level facilitators

#### Theme 1: Staffing Models.

Effective staffing models emerged as critical facilitators for delivering comprehensive PMH care at the organizational level. Participants described staffing frameworks that enhanced PMH services through support roles and dedicated specialists. The inclusion of non-clinical support staff, such as social workers, was widely recognized as valuable in supporting both patients and clinicians. Participants also emphasized the importance of having dedicated mental health specialists within maternity services to enable timely referral and targeted support.

#### Theme 2: Hospital Services and Infrastructure.

Hospital services and infrastructure, particularly service design, accessibility, and integration of mental health services within hospital environments, were emphasized as key organizational facilitators. Participants described ongoing efforts to develop creative and flexible service models. Integrated care models were viewed as facilitating holistic, patient-centred care across settings. The availability of specialised mental health services within hospitals enabled more timely intervention, while co-located services were perceived to improve efficiency and convenience for patients and providers.

#### Theme 3: Care Coordination and Continuity.

Effective care coordination and continuity were identified as fundamental facilitators of PMH care. Multidisciplinary collaboration across teams enabled shared responsibility and coordinated patient support. Continuity of care across the perinatal period was highly valued, with repeated encounters increasing opportunities to identify emerging mental health concerns. Extended care models, including postpartum follow-ups and outreach, were viewed as important mechanisms for capturing unmet needs beyond routine episodic care.

#### Theme 4: Policy and Structural Support.

Policy and structural support emerged as critical organizational facilitators, highlighting the need for institutional frameworks that prioritise PMH services. Participants emphasized the importance of formal organizational mental health policies and frameworks. Mental health awareness and education for patients, families, and staff were identified as key priorities. Service accessibility initiatives, including integrated care models, helped reduce stigma. Standardised screening systems were seen as essential for consistent identification of PMH concerns. Electronic health records supported privacy and continuity of care, while clear referral guidelines and tracking systems were considered crucial for effective follow-up.

#### Theme 5: Workforce Support.

Workforce support was identified as a key organizational facilitator, encompassing both capacity building and staff wellbeing initiatives. Participants highlighted the importance of accessible mental health resources for healthcare professionals managing perinatal cases. Self-directed learning and access to trusted clinical resources supported professional development. Organizational practices such as adherence to clinical guidelines and supervisory support were also noted. Staff wellbeing programmes were viewed as essential for reducing stigma and encouraging help-seeking among healthcare professionals themselves.

### Political-level barriers

#### Theme 1: Financial and Insurance Constraints.

Financial and insurance-related obstacles were reported as one of the most significant political barriers preventing access to mental health care services. Participants observed that health insurance policies do not cover mental health referrals, therapies, or treatments for all patients, forcing some individuals to pay out of pocket for essential services. This financial burden was described as particularly challenging for those with basic insurance plans or no coverage. High treatment costs were seen as a major deterrent, leading some patients to forgo necessary mental health care.

These challenges were further compounded by healthcare system limitations that restrict access to affordable options for uninsured patients. Participants highlighted the need for reduced fees and the introduction of hospital-based exemptions, particularly for individuals without comprehensive insurance coverage, to mitigate financial barriers to perinatal mental health care.

### Political-level facilitators

#### Theme 1: Policy-Level Investment in Capacity and Access.

Beyond individual and organizational efforts, participants emphasized the importance of policy-level investment in capacity and access as a fundamental driver for improving PMH care. Government-led training initiatives were identified as key mechanisms for strengthening workforce capacity, with ongoing programs described as expanding across public and private healthcare settings. In addition, increasing insurance coverage and reducing financial barriers were emphasized as critical strategies for improving equitable access to mental health services. These policy-driven measures reflect systemic efforts to strengthen PMH care through enhanced education, affordability, and access.

## Discussion

**Fig 1:**
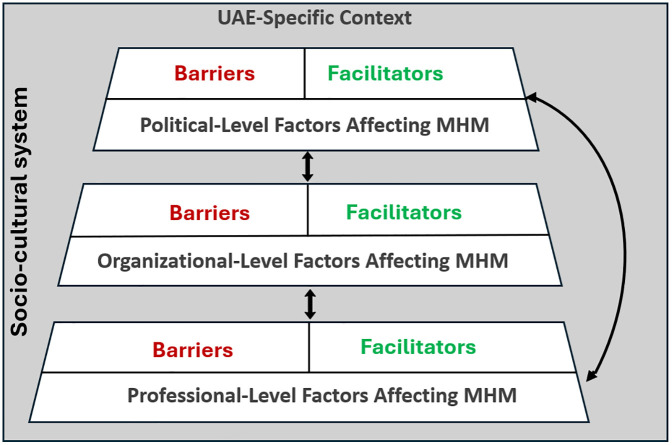
Multi-Level Conceptual Framework of Determinants in Perinatal Mental Health Care in the UAE. The framework highlights political-, organisational-, and professional-level barriers (red) and facilitators (green) to mental health management (MHM) identified by healthcare professionals, illustrating their bidirectional interactions and systemic influences. The socio-cultural system is positioned as a mediating factor across all levels.

This study examined the professional, organizational, and political-level barriers and facilitators to implementing PMH practices in maternity settings in the UAE. It aimed to understand healthcare professionals’ perspectives and identify actionable recommendations to enhance the delivery and integration of PMH care within existing maternity services. The findings reveal multi-level challenges, as illustrated in Fig 1. At the professional level, gaps in PMH awareness, competencies, training, and interprofessional collaboration were evident. Organizational barriers included fragmented services, inconsistent protocols, and staffing shortages, while political-level constraints encompassed limited insurance coverage. Key facilitators included professional development initiatives, integrated care models, multidisciplinary collaboration, culturally sensitive practices, and supportive policies prioritizing PMH. Collectively, the results highlight a systemic underpreparedness, where limited exposure to PMH cases, low self-efficacy, and role-based avoidance restrict providers’ ability to deliver timely care. Organizational fragmentation and unclear referral pathways further delay diagnosis and treatment. Conversely, ongoing training, integrated psychiatric support, and strong provider–patient rapport help overcome these barriers, demonstrating that coordinated, culturally competent systems improve care outcomes. The providers’ narratives revealed the bidirectional interactions between these multi-level factors. Furthermore, the socio-cultural system was positioned as a mediating factor across all levels, as seen in the influence of family and stigma.

At the professional level, gaps in PMH competencies, training, and interprofessional coordination constrained timely identification and management of PMH concerns. Limited clinical exposure, low self-efficacy, and role-based avoidance shaped how providers engaged with PMH care, particularly in the absence of clear referral pathways and shared responsibility. These findings align with international evidence showing that PMH care often falls outside the perceived scope of non-specialist maternity providers when training and systems support are insufficient [26,27]. Similar challenges related to role clarity and referral confidence have been reported among nurses, midwives, and primary care providers in other contexts [28–30]. suggesting that professional-level barriers are closely tied to how care pathways are organised rather than to individual motivation alone. While training initiatives can improve knowledge and attitudes, sustained practice change requires organisational endorsement, protected time, and formalised protocols that enable providers to act with confidence [31].

Interprofessional collaboration emerged as a central mechanism for addressing these professional-level constraints. Clearer role delineation, shared responsibility across disciplines, and routine collaboration were viewed as essential for reducing uncertainty and communication breakdowns previously documented in maternity care settings [32]. Exposure to diverse professional and cultural perspectives further strengthened providers’ capacity to deliver responsive care, reinforcing calls to integrate cultural competence within PMH training and service delivery [33]. Core professional attributes,such as empathy, compassion, and person-centred communication, remained important enablers, but their effectiveness was strongly mediated by organisational conditions that either supported or constrained their application in practice [33,34].

At the organisational level, the findings echo World Health Organization guidance emphasising the need for system-level reforms to ensure accessible, coordinated, and equitable PMH services [35]. Barriers such as inconsistent screening practices, unclear referral pathways, staffing shortages, and fragmented service provision are widely reported across healthcare systems [36–38]. In contrast, facilitators identified in this study, including integrated care models, multidisciplinary teamwork, and culturally adapted service approaches, align with evidence demonstrating that combining standardisation with contextual flexibility enhances PMH service effectiveness [39–41]. Organisational investment in workforce development, workflow integration, and stigma reduction was viewed as critical for translating professional capability into consistent service delivery [32,39,40,42].

A distinctive contribution of this study lies in its examination of PMH implementation within the UAE context. The multicultural composition of the healthcare workforce emerged as a significant facilitator, enabling linguistically and culturally responsive care in a setting where expatriate professionals predominate [13]. This diversity represents a structural asset for addressing the needs of a heterogeneous patient population. Conversely, insurance-related barriers reflect the UAE’s employer-based health financing model, where limited coverage for mental health services, particularly under basic plans, creates inequities in access, disproportionately affecting migrant populations [15–17]. These findings highlight how workforce strengths can be undermined when policy and financing structures fail to support equitable access to services.

At the political level, financial and insurance constraints were among the most influential barriers shaping PMH care delivery, consistent with global calls for equitable mental health coverage [35,42]. Limited reimbursement, high out-of-pocket costs, and the absence of exemptions delayed help-seeking and restricted continuity of care [43]. Participants emphasised the need for expanded insurance coverage, reduced financial barriers, and explicit inclusion of PMH services within broader health financing frameworks, aligning with international policy recommendations [40,41]. Addressing these constraints requires sustained legislative commitment, targeted resource allocation, and system-level monitoring to ensure implementation aligns with policy intent [12,36].

Taken together, the findings support the MATRIx framework [12] by demonstrating how professional capacity, organisational arrangements, and policy environments interact to shape PMH care delivery. Importantly, this study extends the framework by highlighting the mediating role of sociocultural factors, such as stigma, family dynamics, and workforce diversity, across all levels of implementation. These insights suggest that effective PMH interventions must integrate culturally responsive approaches alongside structural and policy reforms to achieve meaningful and sustainable change.

Several practical implications arise from this analysis. At the professional level, embedding PMH-specific training within maternity services, integrating mental health specialists into routine care, and standardising screening using validated tools such as the Edinburgh Postnatal Depression Scale are essential. Organisational strategies should prioritise integrated care pathways, co-located psychiatric services, and multidisciplinary teamwork. At the policy level, insurance reform and sustained investment in workforce wellbeing are critical to ensuring equitable access and long-term service quality.

This study has limitations. Data were drawn from three UAE cities, which may limit transferability to other regions. As a qualitative study, findings prioritise depth over generalisability, and self-reported data may be subject to social desirability bias. Purposive and snowball sampling may have favoured more engaged providers, and reliance on virtual data collection may have limited non-verbal communication. Finally, the absence of service-user perspectives restricts the findings to provider viewpoints. Future research should incorporate perinatal women’s experiences, examine regional variation in PMH service provision, and evaluate implementation strategies using longitudinal and mixed methods designs.

## Conclusion

This study identifies interconnected professional, organisational, and policy-level factors shaping the implementation of perinatal mental health (PMH) care within maternity services in the UAE. The findings demonstrate that gaps in workforce capacity, fragmented service organisation, and policy and financing constraints continue to limit the consistent delivery of PMH care, despite the presence of enabling professional practices and system-level strengths. Addressing these challenges requires coordinated action across levels rather than isolated interventions. Embedding multidisciplinary, integrated, and culturally responsive models within routine maternity care offers a feasible pathway to strengthening PMH service delivery. Strengthening organisational infrastructure, clarifying referral pathways, and supporting interprofessional collaboration are critical for translating professional capability into sustained practice. At the policy level, mandating routine EPDS screening across antenatal and postnatal services, alongside insurance reforms that ensure comprehensive coverage for perinatal mental health services, represents an immediate and actionable step toward improving access and equity. Without addressing these structural and financing barriers, improvements at the clinical level are unlikely to be sustained.

Overall, advancing PMH care in the UAE requires a systems-oriented approach that aligns workforce development, service design, and policy reform. Leveraging the strengths of the multicultural healthcare workforce within supportive organisational and policy environments is essential for delivering equitable, effective, and sustainable perinatal mental health care for women and families.

## Supporting information

S1 File Inclusivity in Global Research Checklist. Completed PLOS Inclusivity in Global Research checklist detailing the ethical, cultural, and scientific considerations relevant to the conduct and reporting of this study.
(DOCX)
